# Identifying co-targets to fight drug resistance based on a random walk model

**DOI:** 10.1186/1752-0509-6-5

**Published:** 2012-01-19

**Authors:** Liang-Chun Chen, Hsiang-Yuan Yeh, Cheng-Yu Yeh, Carlos Roberto Arias, Von-Wun Soo

**Affiliations:** 1 Department of Computer Science, National Tsing Hua University, HsinChu 300, Taiwan; 2 Institute of Information Systems and Applications, National Tsing Hua University, HsinChu 300, Taiwan

## Abstract

**Background:**

Drug resistance has now posed more severe and emergent threats to human health and infectious disease treatment. However, wet-lab approaches alone to counter drug resistance have so far still achieved limited success due to less knowledge about the underlying mechanisms of drug resistance. Our approach apply a heuristic search algorithm in order to extract active network under drug treatment and use a random walk model to identify potential co-targets for effective antibacterial drugs.

**Results:**

We use interactome network of Mycobacterium tuberculosis and gene expression data which are treated with two kinds of antibiotic, Isoniazid and Ethionamide as our test data. Our analysis shows that the active drug-treated networks are associated with the trigger of fatty acid metabolism and synthesis and nicotinamide adenine dinucleotide (NADH)-related processes and those results are consistent with the recent experimental findings. Efflux pumps processes appear to be the major mechanisms of resistance but SOS response is significantly up-regulation under Isoniazid treatment. We also successfully identify the potential co-targets with literature confirmed evidences which are related to the glycine-rich membrane, adenosine triphosphate energy and cell wall processes.

**Conclusions:**

With gene expression and interactome data supported, our study points out possible pathways leading to the emergence of drug resistance under drug treatment. We develop a computational workflow for giving new insights to bacterial drug resistance which can be gained by a systematic and global analysis of the bacterial regulation network. Our study also discovers the potential co-targets with good properties in biological and graph theory aspects to overcome the problem of drug resistance.

## Background

Drug resistance has been posing an emergent threat to human health and infectious disease treatment. Drug resistance is a natural survival mechanism for bacteria when the cell is exposed to drug exposure. Mycobacterium tuberculosis (Mtb) remained to be one of the leading and widely spread killer infectious diseases. In 2008, estimated 390000-510000 cases of multidrug resistant Mtb recorded in WHO 2010 and this problem is worsened significantly by the emergence of drug resistance under clinical drug used. Up to now, methods employed to tackle the problem of drug resistance are rather arbitrary. Several wet-lab experiments and clinical decisions like rotation of antibiotic combinations, identification of new targets and chemical entities that may be less mutable are being explored to counter this problem by inhibiting the resistance mechanism [1]. However, those strategies are still not effective enough and have so far achieved limited success due to limited knowledge about how the resistance mechanisms are triggered in bacteria upon antibiotic drug treatment [2].

The gene expression depended upon the mechanism of action of the drug in the cell as a consequence of the action through metabolic and regulatory adjustments or triggering drug resistance more explicitly [3]. The high-throughput of microarray technology has led to explosion of data concerning the expression levels of the genes but most of statistical methods such as fold change and t-test identify genes with significant changes. However, based solely on the patterns of variations in terms of the increase or decrease in the expression levels of individual genes, it is in general hard to know the related processes involved in the mechanisms of the drug response and resistance.

Due to the increasing availability of protein interaction networks, network-based analysis provides an opportunity to discover an active (significant) network. The network provides a systems-level view of how genes and their products interact within the cell and explain the biological actions under specific condition. However, one weakness of the protein-protein interaction data is that it contains no information about the conditions under which the interactions may take place which means it is not a real snapshot of the interactions in vivo, but a union of the interactions activated under various conditions. Except protein interaction network supported, the network will be much more biologically insightful if the expression data is incorporated with them. Recently some various network-based approaches [4-8] based on protein interaction networks have obtained much better performance than traditional statistical approaches only based on the gene expression values. They first applied a scoring scheme to evaluate an active level of the network based on the gene expression of each gene or its interactions. In the second step, a search procedure is implemented to find the node connected in a sub-network has a highest score and to form a maximum-scoring sub-network. Due to this kind of the problem is NP-hard, several heuristic or approximate methods such as simulated annealing, locally greedy search and mathematical programming methods were proposed. Ideker et al. first formulated the problem of the active pathway detection, where the scoring function is given by a summation function of all genes' differentially expressed p-value within the sub-networks [9]. Dittrich et al. used an additive function of p-values based on a mixture model [10]. Breitling et al. proposed a method to score active sub-networks in terms of genes' order of their differential expression significance [11]. Sohler et al. searched for active networks by spanning the networks with a given set of seed proteins [7]. Such approaches as the vertex-based methods usually do not further select the active interaction relationships among the identified proteins. However, taking all the interactions among those 'active' proteins detected by those methods is inadequate, because under a particular condition, only a part of the interactions may be active. Guo et al. proposed a novel edge-based scoring to extract an active sub-network related to some investigated gene expression profiles under specific condition [12]. Han et al. calculated the average Pearson correlation coefficients between the hubs and their neighbors in the protein interaction networks [13]. Zhao proposed an integer linear programming (ILP) model to find the signal pathways by utilizing both protein interactions and microarray data [14]. Some edge-based scoring methods use statistical measures such as Pearson correlation coefficient for analyzing the pair relationships which do not work well in the small set of the microarray data and could be also unsuitable to explore true relationships because they are overly sensitive to the expression values.

With the availability of high throughput microarray data and interactome network, it is feasible to address the issue of drug resistance from the system's perspective. Wu et al. integrated those two kinds of the information to present a novel network-based approach to identify effective combination of drugs by comparing the sub-network affected by individual drugs [15]. Typically, the target of a drug inhibits the pathogen or arrests its growth but the resistant machinery is established via certain pathways. A recent idea is to counter the drug resistance that called a "co-target" instead of being the ancillary or secondary targets that have a critical physiological function for the survival of a cell and it helps a primary drug to inhibit the resistance mechanism [2]. Thus, a co-target could be either essential or non-essential but it is necessary to have a strong influence in the resistance network. Raman and Chandra formulated the problem of identifying a co-target as a search for the shortest paths obtained from the bacteria protein interaction network and calculated the "betweenness" attribute of genes to identify the potential co-target based on the gene expression values [2]. Although the shortest path analysis may yield a higher coverage than observed directly neighbors locally from the protein interaction data, this approach considers only a single length which ignores the potential contribution of the other paths with longer length. Due to the small world property of biological network, the shortest path length in a biological network is typically very small and most of the time there will be additional "relatedness" between two gene nodes [16,17]. However, the shortest paths are the only routes of drug resistance and there are some "back-up" ways to make the robustness of the mechanism in bacteria [18]. Without any essential pathways related to the mechanism of drug resistance, Ayati et al. applied balanced network bipartition method to discover the co-targets which separate interaction network into disconnected pieces to effectively disrupt the survival of a bacterium when it has multiple pathways to trigger the drug resistance [18]. However, all of them simply take all the interactions in the public database as the edges and did not take the active interactions under antibiotic drug treatment into consideration.

We apply a computational approach that uses both gene expression data and interactome network to identify the active networks under antibiotic drug treatment. Then, we apply a random walk model to discover co-targets that are highly likely to affect the genes related to the mechanisms of the drug resistance through the main and back-up paths.

## Methods

**Figure 1:**
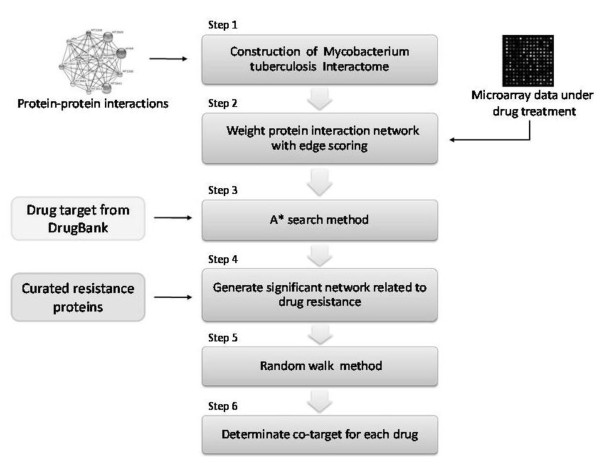
**The overall workflow of our method**.

The overall workflow of our methods consists six steps which is shown in Figure 1. We use interactome network from STRING database in step 1 and assign weight values to the interactions based on the confidence scores and gene expression values from antibiotic drug treatment and control samples in step 2. We present an A* heuristic search method to identify the active networks under antibiotic drug treatment in step 3 and then classify different functional drug resistance pathways using known annotated curated resistance proteins [19] in step 4. We apply random walk method to discover potential co-targets in step 5 and 6.

### Network construction from microarray data and protein-protein interactions database

The microarray data implies gene expression either up-regulated or down-regulated can be revealed in terms of two colored channel in the microarray data representing the intensity of the antibiotic treatment and control samples. The gene expression ratios are calculated as the median value of the pixels minus background pixel median value for one color channel divided by those for the other channel. We extract the median value of the log base 2 of each gene in experimental dataset because the median value of the normalized ratio is much harder to be affected by noise than the mean value. We derive a genome-scale interactome network from STRING database http://string-db.org/ where the interactions extracted from published literature including experimentally studied interactions from genome analysis [20]. The latter is based on well established bioinformatics concepts and methods including structural and functional linkages, genes belonging to a single operon or common neighborhood, pairs of proteins which share metabolites between them and suggested associations based on co-expression, domain fusion or conserved in a number of species [20]. In STRING database, a continuous confidence score is assigned to each interaction which is derived by benchmarking the performance of the predictions against a common reference set of trusted and true associations [20]. A higher confidence score C_uv _is assigned while an interaction between two proteins u and v is supported by more types of evidences.

We formulate an undirected interactome network defined as G(V, E) where the node set V represents protein which is the product of gene *v *(*v*∈V) and edge set E represents the interactions *e *(*e*∈E) in the network. Because the network contains some false positives, we use the absolute value of the expression profile of each gene and the larger value denotes more significant differential expressed genes under drug treatment. We apply the weight to each edge which is defined in Equation (1) as the product of the confidence score C*_uv _*and the sum of the absolute value of gene expression values between two corresponding genes *u *and *v *in the edge.

(1) $$\begin{array}{ccc} w\left(e\right) & = & w\left(u,v\right)=A\left(u,v\right)\\ \; & = & C_{uv}\times \left(\left|Exp_{u}\right|+\left|Exp_{v}\right|\right) \end{array}$$

*Exp_u _*and *Exp_v _*are the average of the gene expression values of node *u *and *v *in the microarray. The higher the expression of the *Exp_u _*or *Exp_v _*denotes the larger differential expression changes of the genes. Then, we use the adjacent matrices A = (a*_uv_*)_n*n _to represent interactome network among *n *nodes where a*_uv _*denotes the weights of interactions between nodes *u *and *v*.

### A* algorithm as heuristic search

In order to study the active networks relevant to drug response and resistance, it is required to define the source nodes to understand the flow of drug actions. DrugBank database http://www.drugbank.ca/ provides drug-related information and also determines the drug targets of an antibiotic drug [21]. Previous studies showed that the metabolic adjustments often occur so as to minimize the effect of inhibition on the particular protein and denoted that multiple proteins in the drug-related functional mechanism may be also targeted [22,23]. According to the effect of such adjustments, it is reasonable to consider the proteins involved in the whole pathway as the source nodes rather than an individual protein [2]. Therefore, we use the drug targets from DrugBank database and the genes associated with the drug-related function as the source nodes for searching active networks.

In the protein interaction network, we try to search the linear or tree type active pathways and then assemble those paths to form the sub-network. However, searching linear paths may expand a large collection of new nodes while traversing new level of tree structure. In order to determine the range of path lengths in the network we would detect, we apply the heap-based Dijkstra's algorithm for each node to get the maximum length of the shortest path of all pairs of nodes in the network [24]. This information shows if any pair of nodes in the network can link to others at most the length and we thus use the length of the longest shortest path as the maximum length in the path searching. Therefore, we assume that the active network extraction issue is a minimum score linear path searching problem with the fixed length [25]. First, we normalize the weight w(*e*) of the edge *e *calculated by Equation (1) to be the range [0-1]. Then, we transfer the larger weight of the edge to be a smaller score and the score of the edge *e *between two corresponding genes *u *and *v *is calculated as score(*e*) = score(*u, v*) = -log(w(*u,v*)). The negative logarithm makes larger weight become smaller score and so on. We define the score of a path as the sum of scores of edges in the path and the formula is defined in Equation (2):

(2) $$score\left(p\right)=\underset{e\in p}{\sum }score\left(e\right)$$

Where score(*e*) is the score of an edge *e *in the path *p*.

To speed up the procedure in search of the minimum score linear path, it needs to prune the unexplored new nodes heuristically. We use the idea of A* search to design a pruning strategy and the heuristic function is to determine the weight of a pathway that reflects significance to some extent. We first run the search procedure 5000 times to determine the scores of all paths in the experiments formed a normal distribution. And then, the error rate based on the standard deviation score*_std_*, minimum score as score*_min _*and an average score of edges as score*_avg _*in the distribution help us to find the optimal pathway in estimating a bound heuristic function h(*x*) for a node *x*. We employ an A* search method that can explore heuristically after searching a fix length *d *in the paths and calculate the weight of a path form root node to the current node *x *as function g(*x*). The overall heuristic function of f(*x*) is defined in Equation (3) for finding a pathway with an optimal (minimum) score.

(3) $$\begin{array}{ccc} f\left(x\right) & = & g\left(x\right)+h\left(x\right)\\ \; & = & score\left(P_{d}\right)+score_{\text{min}}\times \left(l-d\right) \end{array}$$

where *l *means the length of a path, *d *means the length from the source node that we have already traversed in the network, score(*P_d_*) means the sum of the score up to the current node *x *with a length parameter *d*, score*_min _*means the minimum edge score in the network.

Because the lower f(*x*) a node is estimated, the more likely is it to be searched first. We set a bound score for a path *p *with length *l *that is defined as Equation (4) to control the quality of the path we could find:

(4) $$Bound\left(p\right)=\left(score_{avg}-\alpha \times score_{std}\right)\times l$$

α is a constant factor to control the bound, score*_avg _*and score*_std _*means the average score and the standard deviation.

**Figure 2:**
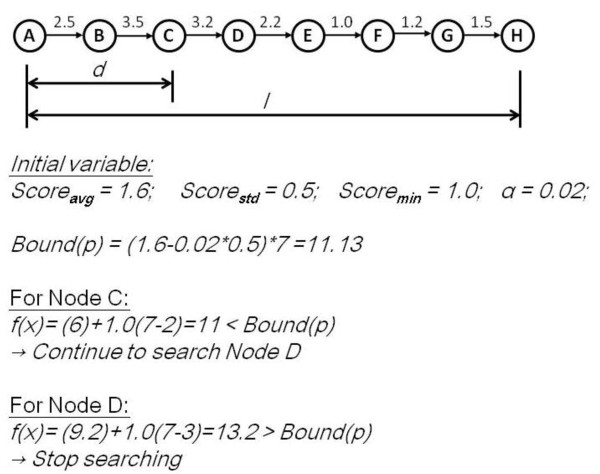
An example for A* searching method.

While we move to the next node through the edge in each search process, we compute heuristic function f(*x*) and compare it with the initially-set bound score. If f(*x*) exceeds the initially-set bound score, we do not expand the node further. For the nodes that are allowed to expand, their children nodes are expanded and their heuristic functions are computed and compared with the bound score again until the search reaches the end node. As the example in Figure 2, we consider finding a pathway with length *l *= 7 from the initial node A to the end node H. First we explore a fix length d = 2 from initial node A that lead us to node C, we start to estimate the score of a path with an additional length of 5 that yields a total weight 11 from current node C. The estimated score of the path is smaller than the bound score 11.13, therefore, we continue to traverse its children. The function of f(x) of current node D is 13.2 and therefore we cannot search into its children. We apply heuristic method to prune the search space instead of exhaustively searching for all the edges in the network.

The known drug resistance genes reported in the previous research further help in classification of the paths [19]. After searching the paths using our heuristic method, we identify the potential drug resistance pathways with at least one curated resistance protein within the paths and assemble them into the active resistance network with active drug resistance gene set G_DR_.

### Random walk to discover co-target

Random walk (RW) is a ranking algorithm which simulates a random walker starts on a set of seed nodes and moves to its immediate neighbors randomly at each step [26]. Each node in the graph is ranked by its probability of the random walker reaching the other nodes and the procedure of the RW model provides the basic idea to estimate the influence from the drug target to the other genes in the weighted network.

### Initial probability for primary drug treatment using RW

Based on the characteristic of RW, we apply it to discover potential co-targets that have the maximum probability to affect the genes related to the drug resistance mechanisms. First, for every node *v *(*v*∈V), we define adj(*v*) which describes the set of nodes *u *with direct interaction with node *v *in the network G, and ws(*v*) as the sum of the weight associated from node *v *to its neighbors *u *in adjacency matrix A, their formal definitions are in Equations (5) and (6), respectively. The transition matrix M for RW is computed using the adjacency matrix A and ws(*v*) and the transition probability M*_uv _*from node *v *to node *u *is defined in Equation (7) where w(*v,u*) is calculated by Equation (1)

(5) $$adj\left(v\right)=\left\{u|\left(v,u\right)\in E\right\}$$

(6) $$ws\left(v\right)=\overset{}{\underset{u\in adj\left(v\right)}{\sum }}w\left(v,u\right)$$

(7) $$\begin{array}{c} M_{vu}=probability\left(v-u\right)\\ \;=w\left(v,u\right)/ws\left(v\right) \end{array}$$

Let *P_0 _*be the initial probability vector constructed in such a way that equal probabilities were assigned to all the source nodes with sum of their probabilities equal to 1. Let *P_s _*be a vector in which a node in the network holds the probability of finding itself in the random walk process up to the step *s*, the probability of *P_s+1 _*can be derived by

(8) $$P_{s+1}=M^{T}P_{s}$$

We plunge the transition matrix M and initial probability vector *P_0 _*into the iterative Equation (8). After certain steps, the probabilities will reach a steady state which is obtained by performing the iteration until the difference between *P_s _*and *P_s+1 _*measured by *L1 *norm falls below a very small number such as 10^-8^. We define the vector *P_reference_(d) *as the reference probability vector represents a steady state probability vector of nodes for the treatment merely by the drug target *d*.

### Discovering potential co-target

A combination of primary drug target and co-target should disrupt network and reduce the emergence of drug resistance thus allowing the main drug to kill the bacteria. The potentially predict co-target by looking at the topological properties of nodes in a drug-treated network in which a co-targeted protein is deleted or inhibited corresponding to remove or hard to reach it [27,28]. It is possible to analyze the consequences on network structure by looking to the variations of the probability of all the other nodes while inhibiting the co-target protein. Given the weighted network of the primary drug treated network, we modify the transition matrix in order to determinate the probabilities of the resulting interactions after deleting or inhibiting the candidate co-targets. It is reasonable to assume the following constraints to specify the new transition matrix M':

(1) To inhibit proteins that are co-targets, the probability of the co-target nodes in the transition matrix should be set to a small value *ε*. In order to represent the inhibition of node in the network, the transition probability from node to the co-target protein should smaller than previous values. Therefore, we set the range of the parameter *ε *from 0 to *TP_min _*which is the minimum probability of the edge in the original transition matrix M.

(2) The sum of the probability of the nodes should be equal to 1, so the probabilities of the rest of nodes must be re-adjusted accordingly if at least one of the edges is set to *ε*.

In order to satisfy the above constraints, let ct(*v*) be a set of proteins whose nodes belong to adj(*v*) which are also co-targets, we have the definition in Equation (9). If ct(v) is an empty set, it denotes that node v does not direct interact with any co-target that we set in the experiment.

(9) $$ct\left(v\right)=\left\{\;u|adj\left(v\right)\wedge u\text{ is a co - target }\right\}$$

For every node *v *in the network, if a node *u *in adj(*v*) belongs to ct(*v*), we reset the probability of walking into a co-target node with a small value *ε *(since it is assumed to be inhibited), else, let the number of the nodes in ct(*v*) as |ct(*v*)| and the sum of the weights of the nodes in adj(*v*) which are not in ct(*v*) as ws'(*v*) defined in Equation (10), we adjust the weight to each node which is not in ct(*v*) based on the weight ratio of w(*v, u*) and ws'(*v*) and one minus the probabilities attributed to co-target nodes as in Equation (11).

(10) $$ws^{\prime }\left(v\right)=\overset{}{\underset{w\in adj\left(v\right)-ct\left(v\right)}{\sum }}w\left(v,u\right)$$

(11) $$\begin{array}{c} M_{vu}^{\prime }=probaility\left(v\to u\right)\\ =\left\{\begin{array}{cc} \varepsilon \; & u\in ct\left(v\right)\\ \frac{w\left(v,u\right)}{ws^{\prime }\left(v\right)}\left(1-\left|ct\left(v\right)\right|\varepsilon \right) & \;u\notin ct\left(v\right) \end{array}\right) \end{array}$$

**Figure 3:**
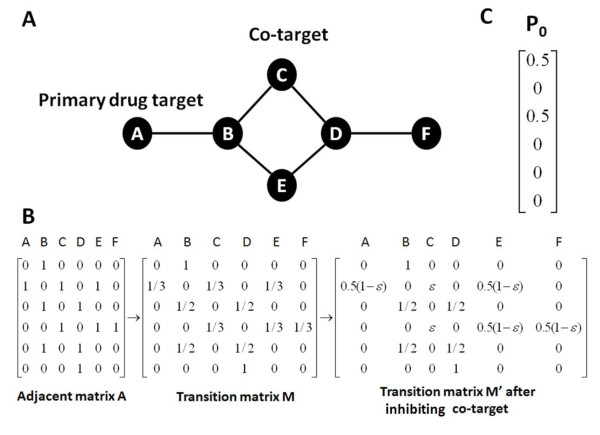
**An example of the transition matrix in the co-target assignment**.

The small undirected network is represented in Figure 3(A) where node A is a primary drug target and all the weights of the edges are equal to one. Figure 3(B) is the adjacent matrix A and entries of the original transition matrix M are calculated according to Equation (7). While we choose the node C to be a co-target, the modified transition matrix M' is calculated according to Equations (9)-(11). Take node B as an example, first we get adj(B) = {A, C, E} and ct(B)={C} from Equation (9) and then we set the probability of M'_BC _and M'_DC _to be *ε *based on Equation (11). The probability of M'_BA _is calculated by

(12) $$\begin{array}{c} M_{BA}^{\prime }=probaility\left(B\to A\right)\\ =\left(\frac{1\;/)\;3}{1\;/)\;3+1\;/)\;3}\right)\left(1-\left(1\right)\varepsilon \right)=\frac{1}{2}\left(1-\varepsilon \right) \end{array}$$

The probabilities of M'_BE_, M'_DE_, and M'_DF _are set in a similar manner. The initial probability *P_0 _*is formed such that equal probabilities are assigned to the nodes that are targeted by the drug and its co-target with the sum equal to 1. In Figure 3(C) example, the initial probabilities for the pair of the primary drug target and its co-target are both set as 0.5 respectively. After certain steps, the probability will reach a steady state to the probability *P_cotarget_(d, t) *under the treatment of the primary antibiotic target *d *and its co-target *t*. Finally, we obtain a function *F(d, t) *which is shown in Equation (13) for every primary drug target and co-target pair. The function *F(d, t) *denotes the relative visit frequency of the gene set G_DR _for the co-target *P_cotarget_(d, t) *against the reference probability *P_reference_(d)*. The larger value of the *F(d, t) *denotes that this co-target has a stronger influence to the active resistance gene set G_DR _with respect to the reference probability where only the drug target is under treatment.

(13) $$F\left(d,t\right)=\underset{g\in G_{DR}}{\sum }P_{cotarget}{\left(d,t\right)}_{g}/P_{reference}{\left(d\right)}_{g}$$

where *P_cotarget_(d, t)_g _*denotes the probability of the g^th ^gene which has the drug resistance in the vector *P_cotarget_(d, t)*. After we calculate each gene *t *in active network using Equation (11), we rank gene sorted by the value of *F(d, t) *and try to identify potential co-targets that tend to have larger impact to G_DR_.

## Results and Discussion

Our interactome networks of Mtb H37Rv extracted from STRING database which contains 3764 proteins with 179920 undirected interactions among them. We extract microarray data from Gene Expression Omnibus (GEO) at NCBI with accession number GSE1642 [29] which contains the most frequently used drugs for the treatment of Mtb, Isoniazid (INH) and Ethionamide (ETA). INH is a first-line drug used worldwide to treat Mtb and ETA is a second line drug. In the experiments, H37Rv treated with 0.2 mg/mL and 0.4 mg/mL INH (in 1 μL/mL EtOH) for 6 h with MIC (0.02 μg/mL) and control cells treated with equivalent amount of EtOH for 6 h. H37Rv also treated with 12 μg/mL and 40 μg/mL ETA with MIC (0.5 μg/mL). It must be noted that it is possible that the high concentration may lead to abnormal expression but there may be also a higher probability to develop drug resistance [2]. INH is known to be an inhibitor of mycolic acid biosynthesis (MAP) and ETA is a structural analog of INH that is also thought to inhibit the same biosynthesis. The MAP model contains 219 reactions and 197 metabolites, mediated through 28 proteins based on the complete and accurate with annotations from the latest literature [30]. After mapping to the microarray data, we use 21 proteins as source nodes for A* search to extract active networks.

### Gene expression analysis in treated Mtb: Isoniazid and Ethionamide

Previous studies [31,32] discovered 71 genes relevant to resistance mechanisms were classified into four types (a) efflux pumps which transport drugs out of the cell, (b) cytochromes and other target-modifying enzymes that cause potential chemical modification of drug molecules, (c) SOS-response and related genes leading to mutations or its regulatory region, (d) proteins involved in horizontal gene transfer (HGT) to import a target modifying protein from its environment. The efflux pump proteins directly pump the drug molecules out of the site of the cell [33,34]. The cytochromes in the resistome are known to modify the drug structure. The proteins classified under the SOS category are believed to be important in mediating mechanisms important for DNA repair and hence in the emergence of mutations that give rise to drug resistance [35]. The proteins in the last category give rise to capabilities of reducing the physiological burden of the drug, typically by degrading the drug in a suitable manner [36-38].

The variation of the gene expression in the microarray data have shown that the lists of genes in fact were either increased or decreased upon exposure to the drug [3]. Table 1 shows the number of the up and down regulated genes belongs to curated resistance proteins microarray data [19]. There are 1920 up-regulated genes, 1806 down-regulated genes and the expression values of the 38 genes are equal to zero in INH samples. On the other hand, 1946 up-regulated genes, 1777 down-regulated genes and the expression values of the 41 genes are equal to zero in ETA samples. In the antibiotic efflux pumps category, there are 7 and 8 up-regulated genes while 10 and 9 genes are in the down-regulated set in INH and ETA samples. In SOS processes category, 5 up-regulated and 4 down-regulated genes are in INH samples while only 1 up-regulated gene and 8-down regulated genes in ETA samples. We take the absolute value of expression value to capture inhibitory activity (negative correlation) as well as activation activity (positive correlation) and we find that 31% (22/71) and 32.4% (23/71) of the genes' absolute expression values are larger than the average of the absolute expression values of all the genes in INH and ETA samples. Two genes with expression values which are larger than two standard deviations are iniA and efpA in both INH and ETA data.

**Table 1:** The number of the up and down regulated genes that belong to the curated resistance proteins in INH and ETA

Drug resistance	INH		ETA	
	**Up (+)**	**Down (-)**	**Up (+)**	**Down (-)**

Antibiotic efflux pumps	7	10	8	9
Hypothetical efflux pumps	2	2	1	3
Antibiotic degrading enzymes	1	0	1	0
Target-modifying enzymes	1	0	1	0
SOS and related genes	5	4	1	8
Genes implicated in horizontal gene transfer (HGT)	1	2	1	2
Cytochromes	15	20	20	15

### The drug response and resistance networks under antibiotic treatment

We extract the paths with the length from three to seven where seven is the maximum length of the all pair shortest paths. We suppose there be a probability density function of the score of the active networks and there be parameters that can maximize the likelihood function to fit the density function. A simple and rapid method to calculate an approximate confidence interval is based on the application of the central limit theorem. We use the mean and variance to calculate the top 5% area as lower limits of the 95^th ^percentile confidence interval in the distribution. We extract 681 and 679 active genes involved in the active networks from INH and ETA samples, respectively. Then, we identify the potential drug resistance pathways under drug treatment where at least one of curated resistance proteins is in the paths and assemble them to the active networks. There are 53 and 235 genes in G_DR _in INH and ETA active resistance networks. The paths to different functional resistance mechanisms for different drugs suggested that a given target may have a higher propensity for eliciting a specific mechanism of resistance [18]. From the functional enrichment point of view, we apply the Database for Annotation, Visualization and Integrated Discovery (DAVID) Gene Functional Classification Tool http://david.abcc.ncifcrf.gov/ to find the annotation for active gene set [39]. DAVID can condense a list of genes or associated biological terms into organized classes of related genes or biological modules by using a novel agglomeration algorithm. This organization is accomplished by mining the complex biological co-occurrences found in multiple sources of functional annotation. We focus on the functional processes and pathway enrichment of the active networks and select 3 out of 14 annotation categories related to the functional pathways and processes including Biological Process and Molecular Function in Gene Ontology (GO) and KEGG pathways. We filter the results that have at least 3 genes in each functional category with P-value < 0.05 and FDR < 0.25 see Additional file 1. Both of the genes treated by INH and ETA would facilitate the survival is seen through fatty acid metabolic and synthase activity and microarray experiments also show up-regulation of the genes involved in these pathways to a great extent [40]. Biotin-requiring enzymes including acetyl-CoA carboxylase, methylcrotonyl-CoA carboxylase, propionyl-CoA carboxylase, and pyruvate carboxylase play essential roles in cell metabolism for the survival and pathogenesis of Mtb [41]. The co-factors facilitate the transfer of CO_2 _during carboxylation, decarboxylation, and transcarboxylation reactions in fatty acid and carbohydrate metabolism. Glycerophospholipid metabolism and ethylbenzene degradation decreasing their gene expression profiles indicate down-regulation in the mechanism of the ETA drug resistance.

**Figure 4:**
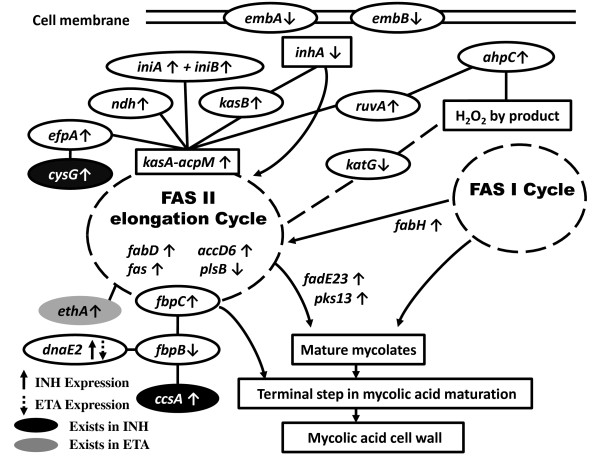
**Part of the active networks treated with INH and ETA**.

We find that fatty acid metabolism activity and membrane metabolism are closely related to the cell wall biosynthesis in both INH and ETA active networks. Mtb has a very complex cell wall which is composed of the cross-linked peptidoglycans linked to arabinogalactans, and mycolic acids [42]. Lipoarabinomannan is presented in the outer layer of the cell envelope which is anchored in the cell membrane [43]. In addition, mycolic acids have a distinctive chemical nature and a lack of mycolic acid synthesis eventually results in loss of cellular integrity and the bacteria death [44]. Therefore, we focus on those processes and cell wall to explain the drug response and resistance mechanism in Figure 4. Nodes are labeled by their gene symbols and nodes in white color denote the overlapping genes in both INH and ETA active networks. Nodes in black color only exist in INH active network and nodes in gray color only in ETA active network. The dash arrow denotes the different gene expression values in ETA samples.

The effect of INH is captured by modifying the interaction of its known target, NADH-dependent enoyl-[acyl-carrier-protein] reductase, inhA (Rv1484) [45]. Fatty acid degradation pathway is activated for the degradation of cell-membrane lipids due to the interruption of their biosynthesis by the inactivation of drug target inhA [45]. We reveal that the mechanism of INH is known to inhibit the biosynthesis of mycolic acid to break the cell wall by disrupting the fatty acid synthesis-II (FAS-II) pathways in Figure 4. The up-regulated FAS-II pathway is closely connected to a similar up-regulated fatty acid degradation pathway assembled by the enoyl-hydratases and acyl-CoA ligases as well as by the putative acyl-CoA dehydrogenase [45]. Gene inhA formed a complex between the kasA-acpM proteins was proposed to be the primary targets in both INH and ETA samples. The kasA genomic consists of five genes in an operon (fabD, acpM, kasA, kasB and accD6) and all of them encode for enzymes that are involved in the fatty acid biosynthesis pathway [46-49]. The NADH dehydrogenase (ndh) gene is bound to the active site of inhA and we also discover that emb proteins (embA, embB, embC) encoded a functional arabinosyltransferase are also involved in the biogenesis of the mycobacterial cell wall. They may play important roles in the cellular homeostasis of the cell and the expressions of these genes activate certain pathways which could link to the resistance. Although ETA and INH are similar to work against the drug treatment, we find that ETA as a thioamide drug which has been shown to be metabolized by a specific fad enzyme, Rv3854c (ethA) and different routes to the FAS-II pathway. The major DNA polymerase dnaE2 participates in DNA repair synthesis and SOS-related genes which is up-regulated in INH active network while it is down-regulated in ETA active network. On the other hand, we assume that Mtb treated with INH has stronger relationship with the SOS process to trigger drug resistance.

Except the global view of the processed in drug response and resistance mechanism, we list the paths with small scores which belong to different functional resistance mechanisms in Tables 2 and 3 where the average value S_avg_(P) denotes the overall score(*p*) divided by the number of genes involved in the path *p*. We observe that the multidrug efflux pumps appear to be one of the major resistance mechanisms in both INH and ETA resistance network. We show that the linear paths with small scores where fadE family are negatively controlled genes that binds to the transcription factors for regulating the expression of fatty acid biosynthesis [46]. Drug resistance related genes efpA, ccsA, cysG and dnaE2 are strongly associated with fadE family which can contribute directly to the emergence of drug resistance. We observe that efpA exists in both active networks and previous experimental observations from a time-kill kinetics study of Mtb showed that the efflux pumps are the predominant mechanism of drug resistance for INH and ETA [49,50]. The expression of the drug transporting genes (efpA, iniA, iniB, cysG, and ccsA) cause pumping out of the active drug from the cell where cysG and ccsA catalyze the NADPH-dependent processes strongly significant in INH active network. Our findings suggest consistency with the recent experimental results and numbers of the proteins refer to the mechanism of the resistance are identified from literature see Additional file 2. We suggest that different drug targets cause different mechanism of the drug resistance and hence different drugs could trigger resistance through different routes.

**Table 2:** Top paths of INH resistance in active sub-networks

Top paths in active sub-networks	S_avg_(P)
**Antibiotic efflux pumps**	

kasB--fabD--kasA--efpA--fbpC2--fbpB--ccsA	1.277
kasB--kasA--fadE24--efpA--fbpC2--fbpB--ccsA	1.327
kasB--kasA--efpA--fadE24--echA18'--fbpB--ccsA	1.348

Antibiotic degrading enzymes	

fabG1--kasB--acpM--kasA--sigC--blaC	1.247
fabD--kasB--kasA--sigC--blaC	1.299
accD4--accD6--kasB--kasA--sigC--blaC	1.331
fabG1--acpM--accA3--fabD--kasA--sigC--blaC	1.345

SOS response	

kasB--kasA--accD6--fadE23--fadE24--echA18'--dnaE2	1.255
accA3--fabD--kasB--kasA--acpM--ruvA--ahpC	1.262
kasB--acpM--accD6--fadE23--fadE24--echA18'--dnaE2	1.284
inhA--kasB--fabD--kasA--acpM--ruvA--ahpC	1.304
inhA--kasA--kasB--accD6--fadE23--echA18'--dnaE2	1.333
fabD--kasB--accD6--fadA2--fadE24--echA18'--dnaE2	1.337

Cytochromes	

inhA--kasB--kasA--acpM--gpdA1--fbpB--ccsA	0.961
kasA--kasB--accD6--fadE23--fadD11--fbpB--ccsA	0.964
kasA--kasB--accD6--fadE23--echA7--fbpB--ccsA	0.975
kasA--kasB--accD6--fadE23--echA5--fbpB--ccsA	0.998
kas--acpM--kasB--fabD--atpD--ctaD--aceE	1.214
kasB--acpM--kasA--fabD--ctaD--aceE	1.247
kasA--kasB--acpM--accA3--fabD--ctaD--aceE	1.248

**Table 3:** Top paths of ETA resistance in active sub-networks

Top paths in active sub-networks	S_avg_(P)
**Antibiotic efflux pumps**	

birA--accA3--fabD--kasA--Rv0342--Rv0341--chaA	1.268
birA--kasB--accD6--fadE23--fadE24--efpA--Rv3570c	1.273
birA--fabD--kasB--acpM--kasA--efpA--Rv3570c	1.359
birA--kasB--fabD--acpM--kasA--Rv0342--Rv0093c	1.364
birA--kasA--kasB--Rv2248--Rv0342--Rv0341--chaA	1.367

Target-modifying enzymes	

fabD--kasB--kasA--acpM--Rv1988--Rv1987--Rv1875	1.402

### The variations of F(d, t) while setting different values of the parameter epsilon

**Figure 5:**
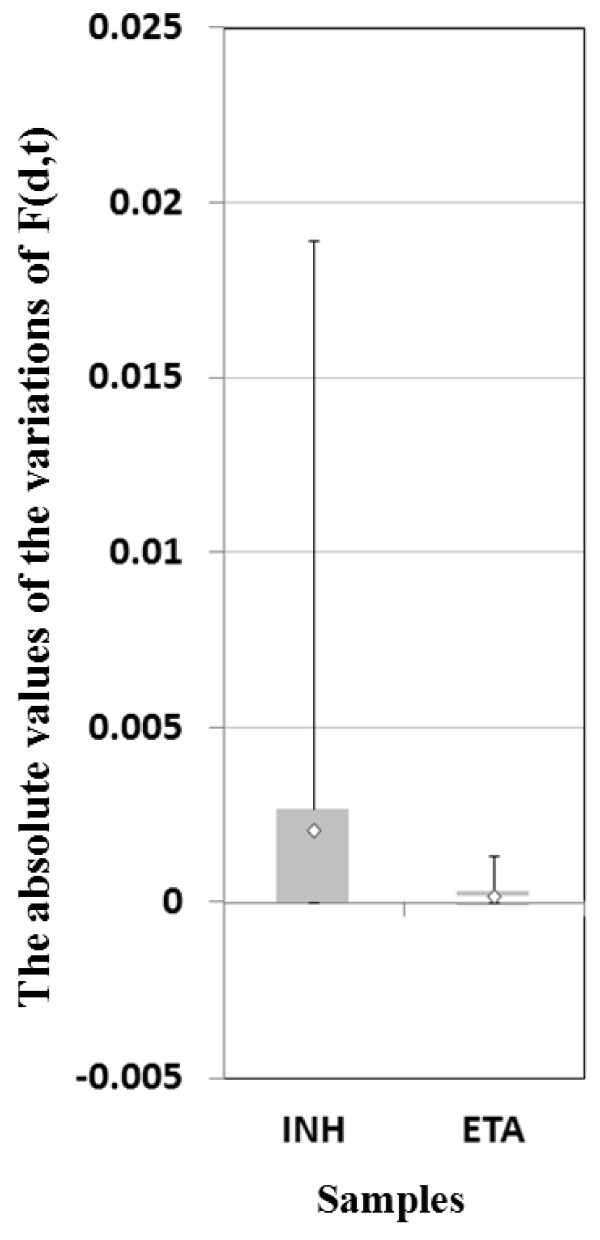
**The absolute values of the variations of F(d, t) among different values of the parameter epsilon in INH and ETA samplesTables**.

In the experiments, TP*_min _*in INH and ETA samples are both 0.0025. We run our method using the values of the parameter ε between 0 and 0.0025 with step 0.0005 and get the set of the probabilities of the nodes calculated by Equation (13). Then, we show the absolute value of the variation of probability of each gene among different values of the parameter ε in INH and ETA samples in Figure 5. The results in both INH and ETA samples have small mean and standard deviation and also denote that the range of the parameter ε we set does not affect the probability of each gene.

### The potential co-targets discovered by random walk model

The unusual multimethyl-branched fatty acids in Mtb are important for the resistance processes of antibiotic resistance, pathogen survival, and virulence [51-53]. Therefore, inhibitors aimed at those processes have high potential to become new anti-tuberculosis therapeutics [54]. We recently suggest the concept of primary drug target and co-target pairs where the co-target could be a key protein in mediating drug resistance. We apply our method to find the potential co-targets with the larger values of the *F(d, t) *in Equation (13). Upon analysis of the potential co-targets only be the up-regulated genes, this indicates that the up-regulated genes seem to have higher influence and play a more important role for a specific purpose. We display 10 and 15 potential co-targets at the top 3% of the function *F(d, t) *in Table 4 and 5, respectively.

**Table 4:** Top 10 co-targets for countering drug resistance under INH treatment

Co-target	Exp	Essential	Annotation	Ref
radA	0.30	Not Essential	DNA repair protein	[55]
fadE10	0.19	Not Essential	acyl-CoA dehydrogenase	[56,57]
Rv2165c	1.05	In Vitro essential	conserved hypothetical protein	[55]
echA17	0.25	Not Essential	enoyl-CoA hydratase	
Rv2137c	1.32	Not Essential	conserved hypothetical protein	
Ffh	0.25	In Vitro essential	signal recognition particle protein	[61-63]
fadE14	0.12	Not Essential	acyl-CoA dehydrogenase	[58]
fadA	0.20	Not Essential	acyl-CoA thiolase	[55]
fadE12	0.15	Not Essential	acyl-CoA dehydrogenase	[56,57]
cysG	0.16	In Vitro essential	multifunctional siroheme synthase	[64]

**Table 5:** Top 15 co-targets for countering drug resistance under ETA treatment

Co-target	Exp	Essential	Annotation	Ref
pgk	0.12	In Vitro essential	phosphoglycerate kinase	[55]
Rv3802c	0.25	In Vitro essential	conserved membrane protein	[55]
Rv1343c	0.37	Not Essential	lipoprotein	[65,66]
Rv1474c	0.03	Not Essential	transcriptional regulator	
Rv0481c	0.08	Not Essential	hypothetical protein	
Rv2455c	0.63	Not Essential	oxidoreductase alpha subunit	
accA2	0.36	In Vitro essential	acetyl-/propionyl-CoA carboxylase alpha subunit	[59]
fadD18	0.05	Not Essential	fatty-acid-CoA ligase	
fadA5	0.45	Not Essential	acetyl-CoA acetyltransferase	[2]
atpB	0.46	In Vitro essential	ATP synthase A chain	[55,67]
Rv2367c	0.28	Not Essential	hypothetical protein	
Rv3355c	0.01	Not Essential	conserved hypothetical protein	
Rv2199c	0.55	Not Essential	conserved membrane protein	
Rv3310	1.38	Not Essential	acid phosphatase	[68]
nadC	0.59	Not Essential	nicotinate-nucleotide pyrophosphatase	[69]

Gene radA, Rv2165c, fadA, pgk, Rv3802c, and atpB are all annotated as potential targets in a comprehensive target identification pipeline including a network analysis of the protein-protein interactome, a flux balance analysis of the reactome, experimentally derived phenotype essentiality data, antibiotic resistance, sequence analyses and a structural assessment of targetability [55]. Large numbers of the enzymes responsible for fatty acid metabolism are also profiled in both cell wall and membrane fractions and our results observe several potential co-targets belong to FadEs, acyl-CoA and enoyl-CoA which are related processes on the fatty acid breakdown in Table 4[56,57]. One of the fadEs, fadE14 has shown the distinct homology to proteins involved in lipid metabolism [58]. Previous studies have shown that acetyl coenzyme A (acetyl-CoA) carboxylase is a key bacterial component in ATPase enzymatic activity and essential protein-protein interaction which catalyzes the first step in fatty acid synthesis and cell growth [59]. One of the AccD5 (5th subunit of acyl-CoA carboxylases (ACCase), has been strongly implicated as one of the essential ACCases important for cell envelope lipid biosynthesis and also identified as inhibitor [60]. The other acetyl-CoA carboxylase-related proteins involved in the biosynthesis of unique cell wall lipids may be explored as possible targets for new drug targets. The conserved secretion (Sec) pathways perform the protein export which is essential participating in inserting integral membrane proteins into the cytoplasmic membrane for virulence with the help of the signal recognition particle (SRP) [61,62]. We show that potential co-target Ffh is a SRP subunit which is associated with adenosine triphosphate (ATP) binding cassette transporter type of proteins are known to be involved in the efflux of drugs in bacterial systems [63]. DnaE2 polymerase response is up-regulated in INH active network and it is strongly associated with error-prone DNA repair such as radA. SOS response is significant up-regulated in INH samples therefore our method suggest that inhibition of the DNA repair genes may reduce the survival of the bacteria. Although Rv2137c is a hypothetical protein, it strongly interacts with the plasma membrane proteins lipoproteins, adrenodoxin oxidoreductase and cell wall processes which is annotated in STRING database [20]. The potential co-target cysG in INH active network is strongly related to the efflux pump processes and efflux inhibitor is also annotated in potential targets to combat antibiotic resistance [64].

In Table 5, we discover Rv1343c (lprD) which was previously shown to be cell wall-associated by proteomics and it could be a specific inhibitor to counter ETA resistance [65]. Lipoproteins such like lprD carry out important functions efficiently at the membrane aqueous interface and its biosynthetic pathway is also essential for bacterial viability [66]. Bacteria may be inherently resistant with a particular type of cell wall structure with an outer membrane that establishes a permeability barrier against the antibiotic. Previous works denoted that FadA5 appears more times in the shortest paths and also considered it as a hub in the resistance pathways [2]. ATP hydrolysis inhibitor such as atpB would inhibit the generation of cellular energy and it would play a role in the bacteria's defence against cell damage. TMC207 (formerly known as R207910) specifically inhibits Mtb by inhibition of ATP synthesis was recently reported to have a potent and selective anti-mycobacterial activity in Phase II clinical trials as a second-line TB drug [67]. Our identification of mycobacterial acid phosphatase may a new target preventing the establishment of intracellular in Mtb and this finding is also characterized in previous papers [68]. Boshoff et al. studies demonstrated that the genes in the NAD biosynthetic pathway offers an important view to understand of a resistance mechanism and also can be an attractive potential targets [69].

The essential genes are required to sustain cellular life and they make excellent to be drug targets. In Table 4 and 5, there are ~30% of genes in the co-targets set are essential based on the essentiality information retrieved from TB [70] and DEG v5.0 database [71]. It is noticeable that those essential genes in the co-target sets have fundamental roles in cell and inhibition of each of them is harmful for cell survival. On the other hand, the selected co-targets have good properties in biological and graph theory aspects. The biological validation for the predicated results from our method is difficult, but it turns out that some of our predicted results had been reported in the public literature for validation. According to our results, we focus on the strategies adapted by Mtb to counter the drug resistance by assessing mechanical barrier and ATP energy. Mechanical barrier is one of the protective strategies employed by Mtb against drug resistance by the alteration of the lipoglycans or fatty acid processes. ATP energy-based and cell wall-related inhibitors may make bacteria loss energy to survive against cell damage.

## Conclusions

To tackle the problem from a drug resistance perspective, it is essential to understand the molecular mechanisms by which bacteria acquire drug resistance using a network-based approach. We develop a computational workflow for giving new insights to bacterial drug resistance which can be gained by a systems-level analysis of bacterial resistance networks. In our approach, we utilize information on STRING database and expression data to construct a weighted network and to decipher the active networks related to drug resistance using A* search method. We discover that genes in both INH and ETA active networks would facilitate survival related to trigger the processes in cell wall, fatty acid metabolism and synthesis, and NADH-related processes. Efflux pumps appear to be the major mechanisms of resistance under INH and ETA drug treatment in Mtb and SOS response is significant involved in INH active network. Several correlations for the predicted resistance paths corresponding to the experimental data are available in literature suggesting that information flows through the identified routes are probable and biologically significant.

We globally identify the potential co-targets which have higher probabilities to affect the genes related to the drug resistance mechanism through the main and back-up paths using our random walk model. Those co-targets related to lipo-rich membrane, ATP energy and cell wall-related processes for countering drug resistance. Knowledge of the active networks under drug treatment help us address more systematic and novel ways to discover the potential co-targets with good properties in biological and graph theory aspects for overcoming the problem of drug resistance. It is important to bear in mind that studies on a theoretically derived network may have some limitations due to missing out some important interactions have not been identified. In the future, the genome of the drug-resistant strain and non-drug-resistant strain can be compared to identify genes which also worth considering as significant feature for co-targets in the sequence level. Inhibition of the primary target and the co-target simultaneously seems to be a feasible and novel way to overcome the problem of drug resistance for other diseases.

## Competing interests

The authors declare that they have no competing interests.

## Authors' contributions

LC participated in algorithm design, performed program and statistical analysis. HY carried out the design of the workflow, algorithm and molecular studies and drafted the manuscript. CY participated in program design. CRA carried out algorithm design. VW participated in its overall design and coordination of the research and helped to draft the manuscript. All authors read and approved the final manuscript.

## Supplementary Material

Additional file 1 **The functional enrichment analysis of the genes treated by INH and ETA using David toolkit with p-value < 0.05 and FDR < 0.25**. File name: additionalfile_1.pdf. We do the functional enrichment analysis of the genes in the drug resistance network treated by INH and ETA using DAVID toolkit. The first column denotes the functional terms. The second and third columns denote the p-value and FDR values. The last column "Cum(exp)" gives the cumulative expression value of the genes annotated in the functional term. Click here for file

Additional file 2 **Known genes related to the drug response and resistance under INH and ETA treatment**. File name: additionalfile_2.pdf. We list the drug response and resistance genes which have received considerable attentions from public literature. Click here for file
