# Supplementary material for: Identifying co-targets to fight drug resistance based on a random walk model
Source: BMC Syst Biol. 2012 Jan 19;6:5. doi: 10.1186/1752-0509-6-5 (PMC3296574; doi:10.1186/1752-0509-6-5)
Supplement: Additional file 1 — The functional enrichment analysis of the genes treated by INH and ETA using David toolkit with p-value < 0.05 and FDR < 0.25. File name: additionalfile_1.pdf. We do the functional enrichment analysis of the genes in the drug resistance network treated by INH and ETA using DAVID toolkit. The first column denotes the functional terms. The second and third columns denote the p-value and FDR values. The last column "Cum(exp)" gives the cumulative expression value of the genes annotated in the functional term. [file 1752-0509-6-5-S1.PDF]

The functional enrichment analysis of the genes in the resistance network treated by INH using David toolkit with p-value < 0.05 and FDR < 0.25

Term	P-value	FDR	Cum(exp)
mtu00640:Propanoate metabolism	3.02E-17	3.01E-14	5.61
GO:0006631~fatty acid metabolic process	2.65E-15	2.54E-12	10.48
mtu00280:Valine, leucine and isoleucine degradation	1.56E-14	1.56E-11	6.21
GO:0004312~fatty-acid synthase activity	3.09E-11	3.27E-08	8.32
GO:0006633~fatty acid biosynthetic process	9.32E-11	8.90E-08	8.39
mtu00071:Fatty acid metabolism	3.49E-09	3.49E-06	4.01
mtu00380:Tryptophan metabolism	4.22E-08	4.21E-05	1.68
GO:0008610~lipid biosynthetic process	6.05E-07	5.77E-04	8.39
mtu00310:Lysine degradation	7.70E-07	7.69E-04	2.32
mtu00632:Benzoate degradation via CoA ligation	7.93E-07	7.92E-04	1.78
mra00380:Tryptophan metabolism	8.94E-07	8.93E-04	1.76
mtu00281:Geraniol degradation	3.79E-06	0.003788	3.84
mtu00620:Pyruvate metabolism	6.00E-06	0.005995	2.60
mtu00650:Butanoate metabolism	1.00E-05	0.010012	2.32
GO:0016885~ligase activity, forming carbon-carbon bonds	9.17E-05	0.09694	2.90
GO:0016053~organic acid biosynthetic process	9.21E-05	0.08786	8.39
GO:0046394~carboxylic acid biosynthetic process	9.21E-05	0.08786	8.39
mra00650:Butanoate metabolism	1.17E-04	0.116775	2.40

The functional enrichment analysis of the genes in the resistance network treated by ETA using David toolkit with p-value < 0.05 and FDR < 0.25

Term	P-value	FDR	Cum(exp)
mtu00281:Geraniol degradation	9.02E-62	1.06E-58	3.02
mtu00624:1- and 2-Methylnaphthalene degradation	2.65E-42	3.11E-39	2.56
mtu00640:Propanoate metabolism	7.40E-42	8.68E-39	6.75
mtu00632:Benzoate degradation via CoA ligation	6.17E-39	7.23E-36	1.76
mtu00280:Valine, leucine and isoleucine degradation	7.19E-38	8.43E-35	8.11
mra00640:Propanoate metabolism	5.96E-37	6.99E-34	6.53
mra00281:Geraniol degradation	4.57E-35	5.36E-32	1.28
mtu00071:Fatty acid metabolism	5.52E-30	6.47E-27	4.74

mtu00903:Limonene and pinene degradation	6.48E-28	7.60E-25	0.40
mtu00380:Tryptophan metabolism	6.21E-24	7.28E-21	2.15
mtu00930:Caprolactam degradation	2.23E-23	2.62E-20	0.17
mtu00310:Lysine degradation	7.80E-23	9.14E-20	2.76
GO:0003995~acyl-CoA dehydrogenase activity	2.96E-21	3.71E-18	5.00
mra00632:Benzoate degradation via CoA ligation	3.53E-21	4.14E-18	2.43
mtu00410:beta-Alanine metabolism	1.29E-20	1.51E-17	2.86
mtu00650:Butanoate metabolism	9.63E-20	1.13E-16	3.10
mra00930:Caprolactam degradation	1.78E-18	2.08E-15	0.34
GO:0006631~fatty acid metabolic process	8.49E-16	1.12E-12	7.79
GO:0004300~enoyl-CoA hydratase activity	1.52E-14	1.91E-11	0.34
GO:0050660~FAD binding	2.93E-13	3.68E-10	4.48
GO:0055114~oxidation reduction	3.05E-13	3.85E-10	10.05
GO:0050662~coenzyme binding	1.51E-09	1.89E-06	4.57
GO:0016836~hydro-lyase activity	1.57E-09	1.96E-06	1.64
mtu00564:Glycerophospholipid metabolism	8.94E-08	1.05E-04	-0.54
GO:0048037~cofactor binding	1.92E-07	2.40E-04	5.76
mtu00642:Ethylbenzene degradation	4.86E-07	5.70E-04	-0.24
GO:0009055~electron carrier activity	6.48E-07	8.13E-04	7.34
GO:0006633~fatty acid biosynthetic process	9.62E-07	0.001215	7.14
GO:0004312~fatty-acid synthase activity	1.04E-06	0.001309	7.73
mtu00072:Synthesis and degradation of ketone bodies	1.11E-06	0.001303	2.14
mtu00620:Pyruvate metabolism	6.86E-05	0.080355	3.53
