# Supplementary material for: Identifying co-targets to fight drug resistance based on a random walk model
Source: BMC Syst Biol. 2012 Jan 19;6:5. doi: 10.1186/1752-0509-6-5 (PMC3296574; doi:10.1186/1752-0509-6-5)
Supplement: Additional file 2 — Known genes related to the drug response and resistance under INH and ETA treatment. File name: additionalfile_2.pdf. We list the drug response and resistance genes which have received considerable attentions from public literature. [file 1752-0509-6-5-S2.PDF]

Known drug response and resistance genes in INH and ETA [1]

Gene	Function	Protein product	Ref.	INH	ETA
Mycolic acid pathway genes					
katG	Catalase-peroxidase activity	Catalase-peroxidase-peroxynitritase	2,3	●	●
furA	Global negative controlling element	Ferric uptake regulation protein	4,5,6	●	●
ahpC	Oxidative stress response	Alkyl hydroperoxide reductase C protein	7	●	●
ndh	Electron transfer	Probable NADH dehydrogenase	8,9	●	●
dfrA	Intermediary metabolism	Dihydrofolate reductase	10,11	●	
inhA	Mycolic acid biosynthesis	NADH-dependent enoyl-[acyl-carrier-protein] reductase	12,13	●	●
acpM	Fatty acid biosynthesis and meromycolate extension	Meromycolate extension acyl carrier protein	14,15	●	●
kasA	Fatty acid biosynthesis and meromycolate extension	3-oxoacyl-[acyl-carrier-protein] synthase	16,17	●	●
fas	Lipid metabolism	Probable fatty acid synthase	18	●	●
plsB	Lipid metabolism	Possible acyltransferase		●	●
fabD	Lipid metabolism	Possible malonyl coA-acyl carrier protein transacylase	19	●	●
fadE	Mycolic acid synthesis	Probable acyl-coA dehydrogenase	20,21	●	●
accD6	Fatty acid biosynthesis	Acetyl/propionyl-coA carboxylase	22,21	●	●
pks13	Synthesis of a polyketide molecule	Probable polyketide beta-ketoacyl synthase	22	●	●
fbpC	Lipid metabolism	Secreted antigen 85	23	●	●
Efflux pump genes (Drug transport)					
iniA	Drug transport	INH inducible protein iniA	24,25,26	●	●

iniB	Drug transport	INH inducible protein iniB	24	●	●
iniC	Transcriptional mechanism	INH inducible protein iniB		●	●
efpA	Export of drugs	Integral membrane efflux protein	27, 28,29	●	●
Rv1747	Transport of drug across the membrane	Conserved transmembrane ATP-binding protein	30,31 ,32	●	
Rv2136c	Unknown	Possible conserved transmembrane protein	33	●	
Rv2459	Transport of drug across the membrane	Possible conserved integral membrane transport protein	34	●	
Rv0849	Transport of drug across the membrane	Conserved membrane transport protein		●	

Reference

[1] Seepe PM, Victor T, Warren R and Louw GE:**Differential Expression of Gene in Clinical Strains of Mycobacterium Tuberculosis in Response to Isoniazid.** thesis. 2011.

[2] Kapetanaki SM, Chouchane S, Yu S, Zhao X, Magliozzo RS and Schelvis JP:**Mycobacterium tuberculosis KatG(S315T) catalase-peroxidase retains all active site properties for proper catalytic function.** Biochemistry. 2005. **44**:243-s252.

[3] Zhao X, Yu H, Yu S, Wang F, Sacchettini JC and Magliozzo RS:**Hydrogen Peroxide-Mediated Isoniazid Activation Catalyzed by Mycobacterium tuberculosis Catalase-Peroxidase (KatG) and Its S315T Mutant.** Biochemistry. 2006. **45**:4131-4140.

[4] Milano A, Forti F, Sala C, Riccardi G and Ghisotti D:**Transcriptional regulation of furA and katG upon oxidative stress in Mycobacterium smegmatis.** J. Bacteriol. 2001. **183**:6801-6806.

[5] Pym AS, Domenech P, Honore N, Song J, Deretic V and Cole ST:**Regulation of catalase-peroxidase (KatG) expression, isoniazid sensitivity and virulence by furA of Mycobacterium tuberculosis.** Mol. Microbiol. 2001. **40**:879-889.

[6] Zahrt TC, Song J, Siple J and Deretic V:**Mycobacterial FurA is a negative regulator of catalase-peroxidase gene katG.** Mol. Microbiol. 2001. **39**:1174-1185.

[7] Guimaraes BG, Souchon H, Honore N, Saint-Joanis B, Brosch R, Shepard W,

Cole ST and Alzari PM:**Structure and Mechanism of the Alkyl Hydroperoxidase AhpC, a Key Element of the Mycobacterium tuberculosis Defense System against Oxidative Stress.** J. Biol. Chem. 2005. **280**:25735-25742.

[8] Lee AS, Teo AS and Wong SY:**Novel mutations in ndh in isoniazid-resistant Mycobacterium tuberculosis isolates.** Antimicrob. Agents Chemother. 2001. **45**:2157-2159.

[9] Vilcheze C, Weisbrod TR, Chen B, Kremer L, Hazbon MH, Wang F, Alland D, Sacchettini JC and Jacobs WR:**Altered NADH/NAD+ ratio mediates coresistance to isoniazid and ethionamide in mycobacteria.** Antimicrob. Agents Chemother. 2005. **49**:708-720.

[10] Argyrou A, Vetting MW, Aladegbami B and Blanchard JS:**Mycobacterium tuberculosis dihydrofolate reductase is a target for isoniazid.** Nat. Struct. Mol. Biol. 2006. **13**:408-413.

[11] White EL, Ross LJ, Cunningham A and Escuyer V:**Cloning, expression, and characterization of Mycobacterium tuberculosis dihydrofolate reductase.** FEMS Microbiol. Lett. 2004. **232**:101-105.

[12] Vilcheze C, Morbidoni HR, Weisbrod TR, Iwamoto H, Kuo M, Sacchettini JC and Jacobs WR:**Inactivation of the inhA-encoded fatty acid synthase II (FASII) enoyl-acyl carrier protein reductase induces accumulation of the FASI end products and cell lysis of Mycobacterium smegmatis.** J. Bacteriol. 2000. **182**:4059-4067.

[13] Vilcheze C, Wang F, Arai M, Hazbon MH, Colangeli R, Kremer L, Weisbrod TR, Alland D, Sacchettini JC and Jacobs WR:**Transfer of a point mutation in Mycobacterium tuberculosis inhA resolves the target of isoniazid.** Nat. Med. 2006. **12**:1027-1029.

[14] Kremer L, Nampoothiri KM, Lesjean S, Dover LG, Graham S, Betts J, Brennan PJ, Minnikin DE, Locht C and Besra GS. **Biochemical characterization of acyl carrier protein (AcpM) and malonyl-CoA:AcpM transacylase (mtFabD), two major components of Mycobacterium tuberculosis fatty acid synthase II.** J. Biol. Chem. 2001. **276**:27967-27974.

[15] Schaeffer ML, Agnihotri G, Kallender H, Brennan PJ and Lonsdale JT:**Expression, purification, and characterization of the Mycobacterium tuberculosis acyl carrier protein, AcpM.** Biochim. Biophys. Acta. 2001. **1532**:67-78.

[16] Bhatt A, Kremer L, Dai AZ, Sacchettini JC and Jacobs WR:**Conditional depletion of KasA, a key enzyme of mycolic acid biosynthesis, leads to mycobacterial cell lysis.** J. Bacteriol. 2005. **187**:7596-7606.

[17] Chen X, Ma Y, Jin Q, Jiang GL, Li CY and Wang Q:**Characterization of the katG, inhA, ahpC, kasA, and oxyR gene mutations in isoniazid-resistant and susceptible strain of Mycobacterium tuberculosis by automated DNA sequencing].** Zhonghua Jie.He.He.Hu Xi.Za Zhi. 2005. **28**:250-253.

[18] Schweizer E and Hofmann J:**Microbial type I fatty acid synthases (FAS): major players in a network of cellular FAS systems.** Microbiol. Mol. Biol. Rev. 2004. **68**:501-17.

[19] Huang YS, Ge J, Zhang HM, Lei JQ, Zhang XL and Wang HH:**Purification and characterization of the Mycobacterium tuberculosis FabD2, a novel malonyl-CoA:AcpM transacylase of fatty acid synthase.** Protein Expr. Purif. 2006. **45**:393-399.

[20] Goyal A, Yousuf M, Rajakumara E, Arora P, Gokhale RS and Sankaranarayanan R:**Crystallization and preliminary X-ray crystallographic studies of the N-terminal domain of FadD28, a fatty-acyl AMP ligase from Mycobacterium tuberculosis.** Acta Crystallograph. Sect. F. Struct. Biol. Cryst. Commun. 2006. **62**:350-352.

[21] Gande R, Gibson KJ, Brown AK, Krumbach K, Dover LG, Sahm H, Shioyama S, Oikawa T, Besra GS and Eggeling L:**Acyl-CoA carboxylases (accD2 and accD3), together with a unique polyketide synthase (Cg-pks), are key to mycolic acid biosynthesis in Corynebacterianeae such as Corynebacterium glutamicum and Mycobacterium tuberculosis.** J. Biol. Chem. 2004. **279**:44847-44857

[22] Portevin D, Sousa-D'Auria C, Montrozier H, Houssin C, Stella A, Laneelle MA, Bardou F, Guilhot C and Daffe M:**The acyl-AMP ligase FadD32 and AccD4-containing acyl-CoA carboxylase are required for the synthesis of mycolic acids and essential for mycobacterial growth: identification of the carboxylation product and determination of the acyl-CoA carboxylase components.** J. Biol. Chem. 2005. **280**:8862-8874.

[23] Wallis RS, Phillips M, Johnson JL, Teixeira L, Rocha LM, Maciel E, Rose L, Wells C, Palaci M, Dietze R, Eisenach K and Ellner JJ:**Inhibition of isoniazid-induced expression of Mycobacterium tuberculosis antigen 85 in sputum: potential surrogate marker in tuberculosis chemotherapy trials.** Antimicrob. Agents Chemother. 2001. **45**:1302-1304.

[24] Alland D, Steyn AJ, Weisbrod T, Aldrich K and Jacobs WR:**Characterization of the Mycobacterium tuberculosis iniBAC promoter, a promoter that responds to cell wall biosynthesis inhibition.** J. Bacteriol. 2000. **182**:1802-1811.

[25] Colangeli R, Helb D, Sridharan S, Sun J, Varma-Basil M, Hazbon MH, Harbacheuski R, Megjugorac NJ, Jacobs WRJr, Holzenburg A, Sacchettini JC and Alland D:**The Mycobacterium tuberculosis iniA gene is essential for activity of**

an efflux pump that confers drug tolerance to both isoniazid and ethambutol. Mol.Microbiol. 2005. **55**:1829-1840.

[26] Colangeli R, Helb D, Sridharan S, Sun J, Varma-Basil M, Hazbon MH, Harbacheuski R, Megjugorac NJ, Jacobs WR Jr, Holzenburg A, Sacchettini JC and Alland D:**The Mycobacterium tuberculosis iniA gene is essential for activity of an efflux pump that confers drug tolerance to both isoniazid and ethambutol.** Mol.Microbiol. 2005. **55**:1829-1840.

[27] Lomovskaya O and Watkins WJ:**Efflux pumps: their role in antibacterial drug discovery.** Curr.Med.Chem. 2001. **8**:1699-1711.

[28] Danilchanka O, Mailaender C and Niederweis M:**Identification of a novel multidrug efflux pump of Mycobacterium tuberculosis.** Antimicrob.Agents Chemother. 2008. **52**:2503-2511.

[29] Doran JL, Pang Y, Mdluli KE, Moran AJ, Victor TC, Stokes RW, Mahenthiralingam E, Kreiswirth BN, Butt JL, Baron GS, Treit JD, Kerr VJ, van Helden PD, Roberts MC and Nano FE:**Mycobacterium tuberculosis efpA encodes an efflux protein of the QacA transporter family.** Clin. Diagn. Lab. Immunol. 1997. **4**:23-32.

[30] Kahnert A, Seiler P, Stein M, Bandermann S, Hahnke K, Mollenkopf H and Kaufman SH:**Alternative activation deprives macrophages of a coordinated defense program to Mycobacterium tuberculosis.** Eur. J. Immunol. 2006. **36**:631-647.

[31] Molle V, Soulat D, Jault JM, Grangeasse C, Cozzone AJ and Prost JF:**Two FHA domains on an ABC transporter, Rv1747, mediate its phosphorylation by PknF, a Ser/Thr protein kinase from Mycobacterium tuberculosis.** FEMS Microbiol. Lett. 2004. **234**:215-223.

[32] Molle V, Soulat D, Jault JM, Grangeasse C, Cozzone AJ and Prost JF:**Two FHA domains on an ABC transporter, Rv1747, mediate its phosphorylation by PknF, a Ser/Thr protein kinase from Mycobacterium tuberculosis.** FEMS Microbiol. Lett. 2004. **234**:215-223.

[33] Braibant M, Gilot P and Content J:**The ATP binding cassette (ABC) transport systems of Mycobacterium tuberculosis.** FEMS Microbiol. Rev. 2000. **24**:449-467.

[34] Gupta AK, Reddy VP, Lavania M, Chauhan DS, Venkatesan K, Sharma VD, Tyagi AK and Katoch VM:**jefA (Rv2459), a drug efflux gene in Mycobacterium tuberculosis confers resistance to isoniazid & ethambutol.** Indian J. Med. Res. 2010. **132**:176-188.
